# Prevalence, morphology, morphometry and associated clinical implications of mastoid emissary veins: narrative review

**DOI:** 10.1590/1677-5449.202300362

**Published:** 2023-07-17

**Authors:** Rajani Singh

**Affiliations:** 1 Uttar Pradesh University of Medical Sciences, Department of Anatomy, Saifai, Etawah, Uttar Pradesh, India.

**Keywords:** mastoid foramen, mastoid emissary vein, skull base, temporal bone, forame mastóideo, veia emissária mastóidea, base do crânio, osso temporal

## Abstract

The mastoid emissary vein connects the posterior auricular vein to the sigmoid sinus and varies in size, number, location, and course, resulting in clinical complications. This study was conducted in response to the vast clinical implications associated with this vein. The aim of this review is to highlight and describe the prevalence, varied morphology, and morphometry of the mastoid emissary vein, how these varied parameters cause clinical complications, and how these can be rectified and avoided. A literature survey was conducted using various databases and different terms related to mastoid emissary vein were used to search the literature. Pitfalls related to surgery in the vicinity of this vein and their remedies were elucidated. The literature search revealed that the prevalence, morphology, and morphometry of mastoid emissary veins vary immensely and are responsible for morbidity and mortality. Pre-operative identification of mastoid veins is thus essential and so multidetector computed tomography of the temporal bone should be scheduled before planning surgery.

## Resumo

A veia emissária mastóidea que conecta a veia auricular posterior ao seio sigmoide pode variar em tamanho, número, localização e curso, resultando em complicações clínicas. O objetivo desta revisão é destacar e descrever a prevalência, variação morfológica e morfometria da veia emissária mastóidea, além de como esses parâmetros causam complicações clínicas e como corrigi-las e reduzi-las. Foram conduzidas buscas em diversas bases de dados utilizando diferentes termos relacionados à veia emissária mastóidea. As armadilhas relacionadas a procedimentos cirúrgicos realizados nas proximidades dessa veia e as respectivas soluções foram descritas. A pesquisa na literatura revelou que a prevalência, a morfologia e a morfometria da veia emissária mastóidea variam imensamente, sendo responsáveis ​​por alta morbidade e mortalidade. Portanto, a identificação da veia mastóidea deve ser realizada no pré-operatório através de tomografia computadorizada multidetectores do osso temporal, antes do planejamento cirúrgico.

## INTRODUCTION

**Figure 1:**
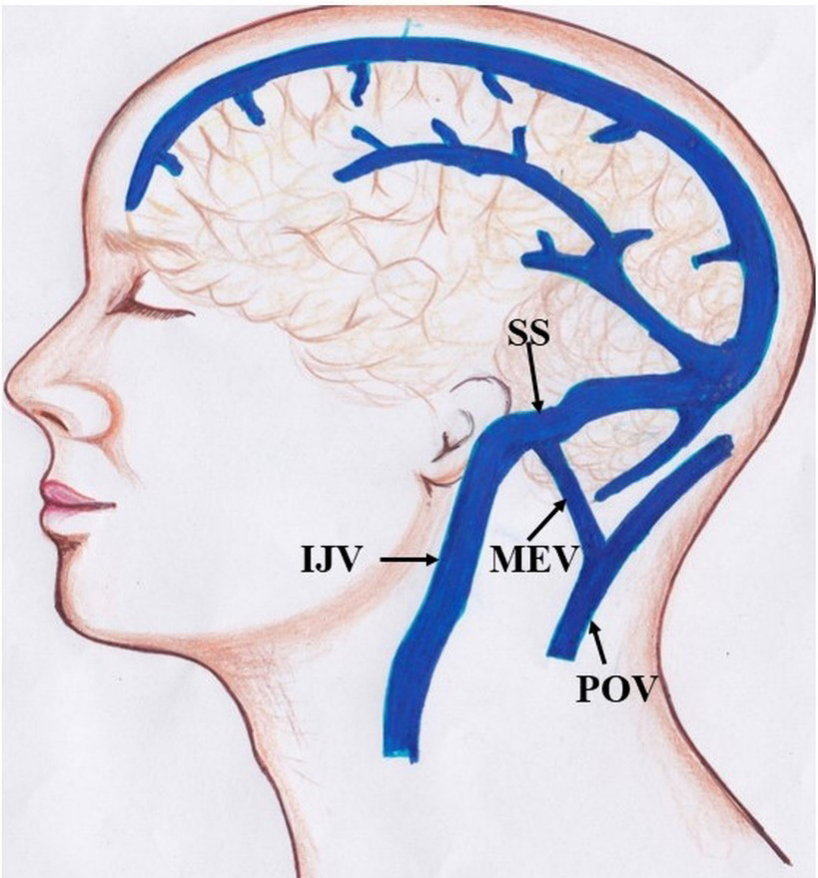
The mastoid emissary vein connecting the posterior auricular vein and sigmoid sinus. SS = sigmoid sinus, IJV = internal jugular vein, MEV = mastoid emissary vein, POV = posterior auricular vein.

Emissary veins course through emissary foramina present in the skull connecting extracranial veins with intracranial venous sinuses and veins. These veins do not contain valves so blood flows through them in both directions.^1^ The emissary veins maintain blood pressure in venous sinuses constant. One of the various emissary veins is the mastoid emissary vein (MEV), also known as vena emissaria mastoidea, which passes through the mastoid emissary foramen (foramen mastoideum) located at or near the vicinity of the mastoid process of the temporal bone. The mastoid emissary vein connects the posterior auricular vein with the sigmoid venous sinus^2^ (Figure 1). The mastoid emissary vein is accompanied by a meningeal branch of the occipital artery known as the ramus meningeus arteriae occipitalis, irrigating the cranial dura mater of the posterior cranial fossa.^3^ In normal healthy individuals, blood flows very slowly in the MEV and is directed from the intracranial venous system to extracranial veins.^4^ However, flow through the MEV increases under conditions of increased intracranial pressure, thereby regulating intracranial pressure by reducing it via increased drainage through the MEV.^5^ In addition to this, during intracranial hypertension, hypoplasia, or aplasia of the internal jugular veins, the MEV may become enlarged, forming a main conduit for cerebral venous drainage and becoming a site of high blood flow and a potential source of massive hemorrhage^5^ during surgical intervention.

Retro-sigmoid craniotomy is the main surgical intervention to reach the posterior cranial fossa for various pathologies in the cranial cavity. The MEV is encountered along the access path during this procedure and injury to it during retro-sigmoid craniotomy may result in massive bleeding. Normally, the MEV is thin and gives rise to minor hemorrhage that can be easily stopped by electric coagulation and bone wax.^6^ However, when the MEV is dilated, as found in various pathological conditions such as vascular malformations, or when the MEV is damaged at its junction with the sigmoid sinus, it is tough to prevent hemorrhage, culminating in shock, infection, and thrombosis.^6^ One case was reported in which injury to the MEV caused so much bleeding that even on applying pressure, blood loss was more than 200 ml in 5 minutes,^7^ which may be due to lack of knowledge of the variability of the MEV with respect to its morphometry and morphology. Thus, knowledge of variations in the course, size, location, and number of the MEV is essential to prevent intraoperative hemorrhage and post-operative complications.

This study was conducted in response to the immense clinical implications associated with the MEV. The aim of this review is to highlight and compile information relating to size, location, and course and correlate these MEV parameters with clinical implications to support neuro and vascular surgeons conducting surgery, facilitating minimum invasion and complications.

## MATERIAL AND METHODS

The study was conducted at the department of Anatomy, UP University of Medical Sciences Saifai, Etawah India. A literature search was carried out using the databases Google scholar, Medline, Scielo, Pubmed, Scopus, ResearchGate, and Wiley Online Library. Standard anatomy text books like Gray’s anatomy and Cunningham’s manual of practical Anatomy were also consulted. Only articles in English were referenced. The terms used to explore literature were, “Emissary veins; clinical significance of emissary veins; Mastoid emissary vein; and clinical significance of mastoid emissary veins”. Information about the importance of the mastoid emissary vein and related pitfalls in surgery at the skull base and lateral skull involving mastoid emissary veins was highlighted.

## LITERATURE REVIEW

The MEV vein varies in incidence, morphometry, course, and number.

### Incidence of MEV

The MEV arises from the inferomedial part of the posterior wall of the sigmoid sinus and courses through the mastoid foramen and then enters the suboccipital venous plexus.^6^ Incidence of the MEV was reported to be 63% in a cadaveric study^8^ and 89% in another study.^5^ The prevalence of the MEV according to the side of the skull is tabulated in Table 1.^5,6,8-14^ It is clear from the table that wide variations are observed in MEV incidence, which may be due to different sample size and methods used by investigators,^6^ but lack of information about this variability causes clinical complications.

**Table 1:** Incidence of mastoid emissary vein on right and left sides of the skull.

**Author**	**Incidence**		**Method**
	**Right side**	**Left side**	
Pekçevik et al.^9^	72.1%	68.6%	Temporal bone CT
San Millán Rúiz et al.^8^	63%		Cadaveric study
Louis et al.^10^	98%	72%	Cadaveric study
Reis et al.^5^	89%		-
Koesling et al.^11^	82%		High resolution CT
Pekcevik et al.^12^	77.7%		CT angiography
Gulmez Cakmak et al.^13^	82.7%	81.4%	MR study
Tsutsumi et al.^14^	89.5%	24.7%	MRI
Zhou et al.^6^	90%		Cadaveric study

### Morphometry of the MEV

The mean diameter of the MEV varies from 0 to 7.3 mm as reported by various researchers (Table 2).^6,7,10,15,16^ Besides transmitting the MEV, the mastoid emissary foramen also transmits the meningeal branch of the occipital artery irrigating the dura mater of the posterior cranial fossa. The dimensions of the mastoid emissary foramen do not therefore reflect the true diameter of the MEV.^6^ Some studies have stated that MEVs with larger diameter are related to diminished size of jugular foramen.^17,18^ In addition to this, large sized MEV carry venous blood from the transverse sinus or sigmoid sinus to the occipital vein and from there to the external jugular vein or to the vertebral vein.^17,18^ The variations in MEV morphometry, including size, impact surgical intervention creating many complications, as vividly described in the clinical significance section.

**Table 2:** Morphometry of mastoid emissary vein.

**Author**	**Mean diameter of MEV**	**Diameter of MEV by %**	**Method**
Louis et al.^10^	3.5 mm, R= 1.1- 5.6 mm	-	Cadaveric study
Kim et al.^7^	1.64 mm	15%= >2.5 mm, 4.3%= >4 mm	Study of skulls
Forte et al.^15^	-	60%= <2 mm, 25%= 2-3.5 mm, 15%= >3.5 mm	Study of skulls
Hampl et al.^16^	1.3 mm, R= 0-7.3 mm	-	Study of skulls
Zhou et al.^6^	1.84 ± 0.85 mm	16.7%= >2.5 mm, 6.7%= > 4 mm	Cadaveric study

MEV = Mastoid emissary vein.

### The intraosseous course of the MEV through the mastoid canal

The course of the MEV through the mastoid emissary canal has been grouped into two types.^6^

Type-I: straight

Type-II: curved

The incidence rates of the straight and curved types were observed to be 57.9% and 42.1% respectively.^6^ The MEV coursing tortuously through the mastoid canal was found to be connected to the diploic vein. During retro-sigmoid craniotomy, MEVs with tortuous course are more likely to be damaged causing hemorrhage. Therefore, the intraosseous course of the MEV should be evaluated preoperatively with CT scanning before planning surgery to avoid damage to this vein.^6^

### Clinical significance of the MEV

The standard incision for retro-sigmoid craniotomy starts in the posterior region of the mastoid process coursing through the asterion and then reaching the lateral part of the posterior neck. In this approach, superficial neck veins including the MEV lie in the path of the incision, increasing the chance of injuring these venous channels. To avoid damage to superficial neck veins, including the MEV, a different type of incision is suggested during retro-sigmoid craniotomy, in which the incision is made 4-5 cm medial to the mastoid process.^5^

The MEV is mostly damaged at the external opening into the mastoid emissary foramen where it is tough to ligate the vein, but this problem can be averted by electrocoagulating and cauterizing the MEV and carrying out bone wax filling to stop bleeding.^6^ However, bone wax may enter the sigmoid sinus causing thrombosis and creating postoperative neurological complications.^6^ This fact is supported by Hadeishi et al.^19^ who observed bone wax in the sigmoid sinus in 7 out of 161 patients undergoing retro-mastoid craniotomy. In addition to this, all these cases had large mastoid foramina needing significant amounts of bone wax to counter hemorrhage.^19^ Therefore, it is suggested that when the dimension of the mastoid foramen is greater than 4 mm as demonstrated by pre-operative CT scan, the MEV should be ligated in addition to using bone wax to stop bleeding during the procedure. To avert complications due to migration of bone wax in the sigmoid sinus, postoperative neurological functions should be evaluated.^6^ However, Rivet et al.^20^, reports that ligation of the MEV may result in venous ischemia and hemorrhage because it is a major source of outflow from the posterior fossa venous sinuses. This fact was supported by a study in which cerebellar infarction occurred in two patients and death in one case due to coagulation of the MEV during skull base surgery.^21^ In the absence of detailed knowledge of the morphometric anatomy of the MEV, especially when its size is 3.5 mm or more, unanticipated hemorrhage may be encountered during mastoidectomy, epitympanectomy, and suboccipital craniotomies,^22^ including formation of epidural and subdural hematoma.^23^ The maximum size reported in literature is about 7 mm.^16^ Such a large MEV size causes massive bleeding. In addition to this, vascular malformations of the internal jugular vein and sigmoid sinus culminating into large sized MEVs are frequent in otitis and labyrinthine dysplasia^24^ and the same can be stated for craniosynostosis surgery and cochlear implantation.^7^ One case report suggests that a dilated MEV can be the single cause of pulsatile tinnitus.^25^ It is thus very important to know the size of the MEV during surgical intervention. The MEV is a well-described landmark for locating the sigmoid sinus and cranial nerves during transcondylar and retro-sigmoid approaches. Information on the MEV is useful for endovascular and posterior fossa surgeries.^26^ The MEV may serve as a conduit for conducting craniofacial tumors and infections into the cranial cavity.^27^ Variable numbers of MEVs have been reported in the range of 0-4.^7^ If there are higher numbers of MEVs, injury to all these veins during surgeries around the mastoid process may culminate into severe hemorrhage that is often difficult to control during surgery if the neurosurgeon is unaware of this numerical variation in the MEV.

## CONCLUSION

The MEV connects the posterior auricular vein with the sigmoid sinus and varies in location, size, number, and course. Knowledge of these MEV parameters is of utmost utility to vascular and neurosurgeons during skull base and lateral skull surgeries including retro-sigmoid, transcondylar, mastoidectomy, epitympanectomy, and suboccipital craniotomies to prevent adverse surgical events due to injury to the MEV. It is very difficult to assess the anatomical features of the MEV preoperatively. Thus, Multidetector computed tomography is suggested shortly pre-operatively to precisely identify the size, number, location, and course of the MEV so as to avoid MEV damage during aforementioned surgical interventions, thus reducing morbidity and mortality. In addition to this, the MEV can be an important channel for transmitting craniofacial tumors and infections into the cranial cavity. This fact should be kept in mind by vascular and neurosurgeons while dealing with pathologies of the cranial cavity.
